# A 129-kb Deletion on Chromosome 12 Confers Substantial Protection Against Rheumatoid Arthritis, Implicating the Gene *SLC2A3*

**DOI:** 10.1002/humu.22471

**Published:** 2013-12-02

**Authors:** Colin D Veal, Katherine E Reekie, Johnny C Lorentzen, Peter K Gregersen, Leonid Padyukov, Anthony J Brookes

**Affiliations:** 1 Department of Genetics, University of LeicesterLeicester, UK; 2 Institute of Environmental Medicine, Unit of Work Environment ToxicologyKarolinska Institute, Stockholm, Sweden; 3 Robert S. Boas Center for Genomics and Human Genetics, Feinstein Institute for Medical ResearchNew York; 4 Rheumatology Unit, Department of Medicine, Karolinska University HosiptalsSolna Karolinska Institute, Stockholm, Sweden

**Keywords:** association, rheumatoid arthritis, SLC2A3, GLUT3, CNV

## Abstract

We describe a copy-number variant (CNV) for which deletion alleles confer a protective affect against rheumatoid arthritis (RA). This CNV reflects net unit deletions and expansions to a normal two-unit tandem duplication located on human chr12p13.31, a region with conserved synteny to the rat RA susceptibility quantitative trait loci *Oia2*. Genotyping, using the paralogue ratio test and SNP intensity data, in Swedish samples (2,403 cases, 1,269 controls) showed that the frequency of deletion variants is significantly lower in cases (*P* = 0.0012, OR = 0.442 [95%CI 0.258–0.755]). Reduced frequencies of deletion variants were also seen in replication materials comprising 9,201 UK samples (1,846 cases, 7,355 controls) and 2,963 US samples (906 controls, 1,967 cases) (Mantel–Haenszel *P* = 0.036, OR = 0.559 [95%CI 0.323–0.966]). Combining the three datasets produces a Mantel–Haenszel OR of 0.497 (*P* < 0.0002). The deletion variant lacks 129-kb of DNA containing *SLC2A3, NANOGP1*, and *SLC2A14. SLC2A3* encodes a high-affinity glucose transporter important in the immune response and chondrocyte metabolism, both key aspects of RA pathogenesis. The large effect size of this association, its potential relevance to other diseases in which *SLC2A3* is implicated, and the possibility of targeting drugs to inhibit *SLC2A3*, argue for further examination of the genetics and the biology of this CNV.

## Introduction

Rheumatoid arthritis (RA) is a chronic autoimmune disease that manifests as inflammation of the synovium and severe joint damage, along with other complications such as cardiovascular disease. It affects approximately 1% of the global population, predominantly women and the elderly, and is treated symptomatically as there is currently no cure. The inflammation of synovial joints in RA leads to hyperplasia of the synovial cells, excess synovial fluid, and the development of pannus (an inflammatory granulation tissue). Persistent synovitis leads to the destruction of articular cartilage and subsequent debilitating bone damage. Environmental factors, such as smoking, play a role in RA risk [Klareskog et al., 2006; Morgan et al., 2009; Silman et al., 1996]. However, around 60% of the overall risk is attributable to genetic factors [MacGregor et al., 2000], approximately one-third of which is conferred by shared epitope HLA alleles within the major histocompatibility complex (MHC) [Gregersen et al., 1987]. A number of other risk loci have been identified, particularly since the advent of SNP-based studies, including *PTPN22* (MIM #600716) [Begovich et al., 2004; Gregersen et al., 2006], *STAT4* (MIM #600558) [Remmers et al., 2007], *C5* (MIM #120900)/*TRAF1* (MIM #601711) [Plenge et al., 2007], and *TNFAIP3* (MIM #191163) [Thomson et al., 2007]. Including recent data from a large meta-analysis of GWAS RA studies, the number of confirmed genetic risk loci is 46 [Eyre et al., 2012; Stahl et al., 2010]. However, these loci contribute relatively modest per locus effect sizes to RA susceptibility (OR ≤ 1.8), leaving much of the genetic risk unaccounted for [as reviewed in Raychaudhuri, 2010]. The remaining genetic risk could be due to other types of variation not routinely investigated such as rare single-nucleotide alleles [Dickson et al., 2010], epigenetic modifications, and copy-number variation (CNV). Latest estimates of CNV suggest that up to 16% of the genome is commonly copy-number variable [Conrad et al., 2010; Itsara et al., 2009; Mills et al., 2011; Redon et al., 2006]. There is increasing evidence for the involvement of CNV in disease susceptibility, not least for autoimmune diseases such as systemic lupus erythematosus [Yang et al., 2007] and psoriasis [Hollox et al., 2008]. Copy-number changes of the *CCL3L1* (MIM #601395) gene have previously been shown to be associated with RA susceptibility and HIV progression [Gonzalez et al., 2005; McKinney et al., 2008].

Previous work by others and us used oil-induced arthritis rat models and linkage analysis to discover RA quantitative trait loci: *Oia1* that contains the MHC genes, and *Oia2* that maps to a 1.2-Mb interval on rat chromosome 4q42 [Jansson et al., 1999; Lorentzen et al., 1998; Ribbhammar et al., 2003]. The rat *Oia2* region shows conserved synteny with human chromosome 12p13.31, which itself resides within a larger RA susceptibility locus [Jawaheer et al., 2003]. We previously demonstrated association between SNPs in this interval and RA in humans [Lorentzen et al., 2007]. Furthermore, assaying SNPs in this region by the dynamic allele-specific hybridization (DASH) genotyping method [Fredman et al., 2004] produced semiquantitative readouts that suggested the presence of structural variation.

We now present the discovery and characterization of a large CNV within the chr12p13.31 interval. Genotyping of this CNV by various methods (laboratory and informatics based) in multiple population samples produced highly consistent evidence that a deletion spanning the *SLC2A3* (MIM #138170) gene confers substantial protection against developing RA.

## Methods

### Samples

Swedish case and control RA samples used in this study have been used for previous investigations [Lorentzen et al., 2007]. All RA case samples fulfilled the American Society of Rheumatology 1987 revised criteria for RA [Arnett et al., 1988]. The Swedish RA cohort was made up of 2,403 patients with RA and 1,269 control samples [Lorentzen et al., 2007]. Controls were collected from the same study area and had similar distribution in age, sex, and residential area. Anti-citrullinated protein antibodies (ACPA) status was available for the Swedish samples of which 64% of cases were ACPA positive. ACPA status was not used to stratify the association data, as there would have been insufficient power to exclude association in ACPA-negative samples. In addition ACPA assays do not detect all ACPA-positive samples (sensitivity 70%–80%) and have a false positive rate of between 4% and 12%. The UK RA case group comprised 1,846 RA case samples, and the UK control group comprised 7,355 samples from the 1,958 British Birth cohort collection as has been previously described by the Wellcome Trust Case Control Consortium [Wellcome Trust Case Control Consortium, 2007]. The WTCCC also assessed this sample for population stratification and only a small number of genomic regions exhibited detectable stratification across a NW/SE divide. The CNV examined in this study did not reside in any of these intervals. The US RA collection consisted of 1,967 cases and 996 controls. The RA case subjects were enrolled from across the United States as part of the North American Rheumatoid Arthritis Consortium (NARAC) collections I and II [Gregersen et al. 2009; Plenge et al. 2007], and all subjects either met the 1987 American College of Rheumatology criteria for diagnosis of RA [Arnett et al., 1988]. As reported previously, controls were obtained from a local New York cohort [Mitchell et al. 2004], and matched to cases using ancestry-informative markers, as described previously [Gregersen et al. 2009; Plenge et al. 2007].

### Oligonucleotide-Array CGH

Oligonucleotide-array CGH was performed by Nimblegen Inc. using 2-μg of DNA on a microarray chip of 152,452 probes spanning 3.5 mb of chr12p13.31. Log_2_ ratios of each of the five pairs of DNA samples were averaged over 500-bp intervals.

### Human Genome Project Trace Archive and BLAT Characterization of Region

Human genome project (HGP) sequencing traces were downloaded from the NCBI Trace Archive (http://www.ncbi. nlm.nih.gov/Traces/) and aligned to the reference genome (build NCBI36/hg18, March 2006) using the GSAssembler V2 software (Roche, Burgess Hill, West Sussex, UK).

### CNV Genotyping Using the Paralogue Ratio Test

The paralogue ratio test (PRT) was selected as it has been demonstrated to be robust in other studies, more reliable than qPCR and has low-DNA quantity requirements [Aldhous et al., 2010; Armour et al., 2007; Cantsilieris and White, 2013; Fode et al., 2011; Hollox et al., 2008]. Assay P1 (primers P1F: 5′-TATTGCACCTTAACCTCTCCAGC-3′ and P1R: 5′-CTCACTTCCATACAGCTCTACG-3′) amplifies two products, one within the 3′ untranslated region of *SLC2A3* (chr12:8073299–8073582), and one within the equivalent region in *SLC2A14* (chr12:7966286–7966484). Partial PRT (pPRT) is a modification of PRT that uses three primers in each reaction; one primer is matched to both targets, and the two remaining primers are each uniquely matched to one target. pPRT primers used are listed in Supp. Table S1. PCR reactions were performed on either 384 or 96 well microtiter plates, case and controls were intermixed on these plates. Genetic association studies can be susceptible to bias resulting from batch effects due to DNA preparation, interlab handling differences, and DNA quality [Clayton et al., 2005; Ionita-Laza et al., 2009]. We have previously performed in-depth investigations into causes of these biases, in particularly how they affect PRT [Veal et al., 2012]. We have utilized methods developed as part of that research to minimize any such effects, if they were present: PCR reactions contained 10 ng DNA, 1× buffer B (Kapa Biosystems, Woburn, MA), 2 M betaine (Sigma-Aldrich, Gillingham, Dorset, UK), 0.2 mM dNTP (Roche), 0.15 μM each primer, and 0.02 U *Taq* DNA polymerase (Kapa Biosystems). PCRs were performed in an MBS 0.2G thermal cycler (Thermo Scientific, Waltham, MA) as follows: 98°C for 1 min; 35 cycles of 98°C for 15 sec, annealing temperature for 15 sec and 72°C for 1 min, followed by a final extension carried out at 72°C for 5 min.

AB1 PCRs were used to directly assay recombination at the AB1 sequence. Primers AF and UR detected P1[B] deletions; each 20 μl reaction contained 20-ng DNA, 1× 11.1× buffer (0.49 M Tris–HCl pH8.8, 0.12 M [NH_4_]_2_SO_4_), 0.05 M MgCl_2_, 77 mM β-mercaptoethanol, 5 μM EDTA pH8.0, 11.1 mM each nucleotide (dATP, dCTP, dGTP, and dTTP), 1.3 mg/ml bovine serum albumin), 0.3 μM each primer, and 0.1 U/μl *Taq* DNA polymerase (Kapa Biosystems). Cycling conditions were: 96°C for 5 min followed by 35 cycles of 96°C for 30 sec, annealing temperature for 20 sec, and 68°C for 4 min. Primers BF and UR were used to investigate P1[B] duplications; each 20-μl reaction contained 20-ng DNA, 1× FastStart High Fidelity Reaction Buffer (Roche), 0.2 mM dNTPs, 0.4 μM each primer, 0.05 U/μl FastStart High Fidelity Enzyme (Roche). Cycling conditions were: 95°C for 2 min, followed by 35 cycles of 95°C for 30 sec, annealing temperature for 30 sec and 72°C for 4 min.

PCR products were separated on 300 ml 2% (w/v) Seakem LE agarose gels (Lonza, Basel, Switzerland) in 1× Tris-Borate-EDTA buffer by electrophoresis. Electrophoresis was performed at 200 V for 45 min. Gel images were captured with the GBOX gel documentation system (Syngene, Cambridge, UK), and signal intensity data for each product were extracted using the GeneSnap software (Syngene).

### Computational Genotyping of the CNV from GWAS Data

PennCNV uses a hidden Markov model utilizing multiple sources of information, including allelic ratio distribution and intensity data, to genotype CNVs in SNP genotyping data [Wang et al., 2007]. In this study, 1,971 Swedish samples had been genotyped using the Illumina Infinium HapMap 300 SNP chip (Illumina, San Diego, CA) and 2,963 US samples that had been genotyped by the Illumina Infinium HapMap 370 and 500 chips (Illumina) as previously described [Gregersen et al., 2009; Plenge et al., 2007]. For all these samples, CNV genotypes were called using the standard PennCNV settings including adjustment for GC waves. As the accuracy of PennCNV is dependent on the size of the CNV, number of SNPs, and quality of SNP genotyping, we plotted the B allele frequency (BAF) deviation from expected values for heterozygous SNPs against mean Log R ratio (LRR) across the CNV to visually assess the clustering of CNV calls (Supp. Fig. S1). For variant samples, there is a clear distinction from normal samples indicating that PennCNV can accurately call this CNV. This is seen particularly for the deletion samples in which there is no overlap with normal samples. For the Swedish samples, P1 genotyping was available for 1,475 of the samples genotyped by PennCNV. For the US samples, for which whole-genome chip-based genotyping data were available, all variant samples detected by PennCNV were inspected visually to confirm the presence of a deletion allele.

### Data Analysis

The ratio of the two products from a PRT or pPRT was calculated by dividing the peak signal intensity of the product from within repeat unit B by the peak signal intensity of the product from within repeat unit A. For assay P1, data for each row of a gel were normalized by multiplying by the reciprocal of the median for each row of samples. Normalized ratios were transformed by log_2_ for further analysis. Samples were categorized according to expected ratios (see *Results*) with boundaries determined by visual inspection of the spread of ratios plotted using the statistical package R [R Development Core Team, 2008]. The significance of any differences in frequency between Swedish case and control samples was determined using the two-tailed chi-squared test on a 2 × 2 contingency table. As our initial data had indicated that the frequency of the P1[B] deletion allele was much lower in the UK and US samples (therefore reduced power to detect association), we combined the US and UK data to maximize power. Given the two populations are distinct both geographically and in method of genotyping, we used the Mantel–Haenszel meta-analysis of the odds ratio. The validity of pooling the odds ratios was confirmed using Woolf's test for heterogeneity.

## Results

### Identification and Characterization of a CNV at chr12p13.31

**Figure 1:**
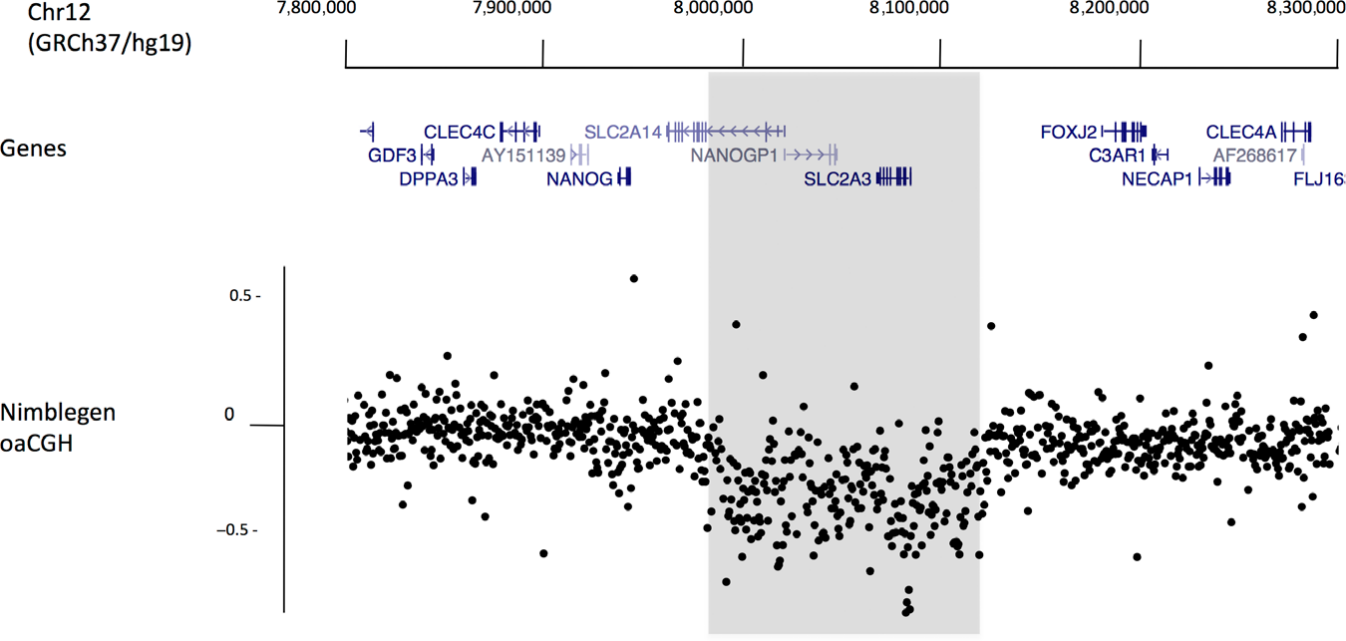
Oligonucleotide-array CGH identifies a CNV on chromosome 12p13.31. Positions of genes are displayed on a UCSC Genome Browser track. Nimblegen oligonucleotide-array CGH data are plotted beneath this track, each point is the average log_2_ ratio of probes in 500-bp windows. A section (>100 kb, shaded) of points deviate from the expected log_2_ of 0 indicating the presence of a copy-number difference between the tested samples.

**Figure 2:**
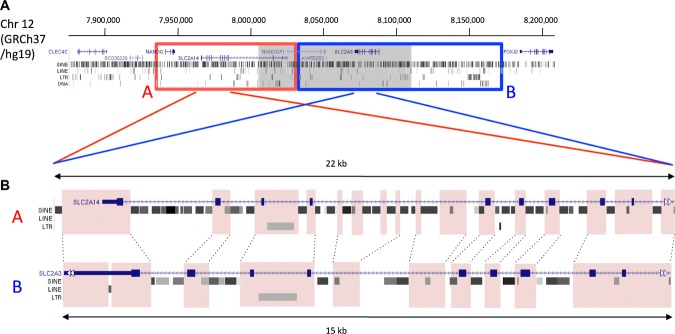
Tandem repeat spans the CNV at chr 12p13.31. A: The position of a tandem repeat at 12p13.31, identified through examination of sequence data, is shown overlaid onto a UCSC genome browser track displaying the genes and repetitive elements within this region. The two repeat units, “A” and “B” are delimited by the red and blue boxes, respectively. The position of the CNV identified by the Nimblegen oligonucleotide-array CGH is shaded in gray and represents a net gain/loss of one repeat unit from the two unit tandem repeat structure. B: The two repeat units are similar in sequence, but have many differences in the repetitive element structure. To illustrate this, equivalent sections of sequence from each unit are presented with the position of genes and repetitive elements taken from UCSC genome browser. Pink shading and dotted lines represent regions of sequence that are similar in both units. It can be seen that differences between the two sections are mainly due to different repetitive elements.

Oligonucleotide-array CGH of the chr12p13.31 region was performed across 10 samples, which were selected based upon DASH genotyping patterns. One oligonucleotide-array CGH experiment, conducted upon a sample pair on chromosome 12 and selected based on DASH genotyping patterns, revealed a large (>100 kb) copy-number change spanning the gene *SLC2A3*, the pseudogene *NANOGP1* and part of the gene *SLC2A14* (Fig. 1). By comprehensive long-range PCR plus next-generation sequencing, mining of public trace archives, and targeted gap-closure experiments via short-range PCR, we established that the 12p13.31 CNV in question entails the gain/loss of one net repeat unit from a two copy tandem duplication (Fig. 2A). The two repeat units, which we termed “A” and “B” (∼100 and 145 kb, respectively), have very diverged patterns of repetitive elements, in particular Alu elements (Fig. 2B), but are otherwise around 95% similar at the DNA sequence level (standard deviation 4%, minimum 80%, maximum 100%, interquartile range 92%–98%, 100 bp windows free of repetitive elements). The tandem duplication is seen to be present in other higher primates (Chimpanzee build CGSC 2.1.3/panTro3; Rhesus Macaque build MGSC Merged 1.0/rheMac2) but not in other mammals such as mice (build NCBI37/mm9). From estimated dates of simian divergence and historic periods of Alu expansion, it may be likely that the ancestral duplication event occurred at least 50 million years ago [International Human Genome Sequencing Consortium et al., 2001]. Four known genes reside within the tandem repeat: *NANOG* (MIM #607937) encoding a transcription factor expressed in embryonic stem cells and a key factor in the maintenance of pluripotency [Mitsui et al., 2003], *SLC2A14* (MIM #611039) encoding GLUT14 a glucose transporter expressed specifically in the testes [Wu and Freeze, 2002], *NANOGP1* that is a transcribed but untranslated pseudogene of *NANOG*, and *SLC2A3* encoding GLUT3, a glucose transporter with the highest affinity for glucose among the family of GLUT proteins, which is expressed in various tissues, including chondrocytes, and plays an essential role in embryonic development [Schmidt et al., 2009].

### CNV Assay Development

**Figure 3:**
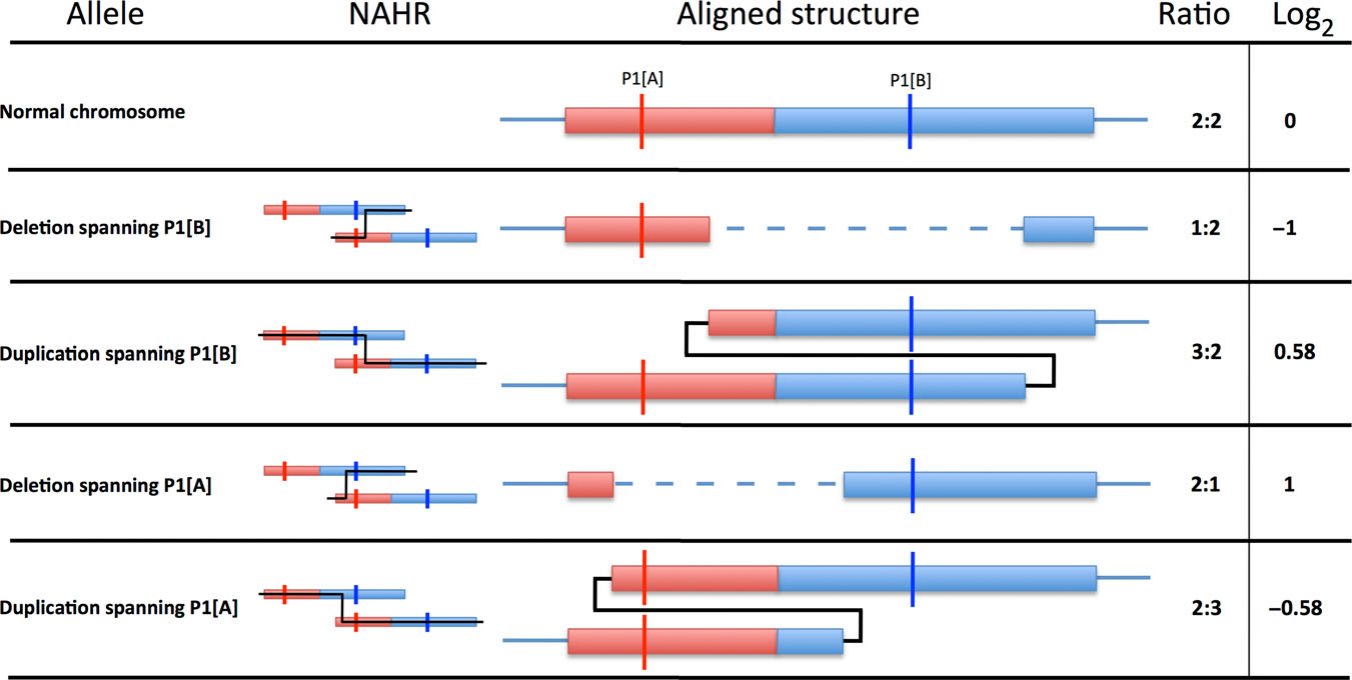
Relative ratios generated by CNV alleles for the P1 assay. Effects of possible CNV alleles on assay P1 are displayed on a diagrammatic portrayal of the tandem repeat. Four theoretical allele types, deletion or duplication spanning P1[A] or P1[B], are displayed below the representation of the tandem repeat on a normal chromosome. The two units of the tandem repeat are presented, “A” in red and “B” in blue. The positions of each of the targets of the P1 assay are marked (P1[A] and P1[B]). An example NAHR event is displayed for each variant allele. The aligned structure column displays the variant chromosome with deleted or duplicated material aligned to the normal chromosome and the effects on each locus of the P1 assay. The ratios of P1B/P1A for a heterozygous individual are displayed for diploid genome ratio, and the log_2_ of the ratio.

To investigate this CNV further, we employed assays based upon the PRT—a method that in typical scenarios uses one pair of PCR primers to coamplify (and hence allow quantitative comparison of) both a test region (whose copy number is being assessed) and a stable single copy reference region [Armour et al., 2007]. However, since the high degree of sequence identity between the two repeat units and the high density of repeat elements in this region precluded the use of a standard PRT, we modified the concept to instead amplify equivalent but differently sized segments from each unit of the tandem duplication. The unit B/unit A ratio of products in this case was then taken to indicate relative changes in copy number between the two units at the sites being amplified, rather than absolute copy-number values. Several assays were initially designed and optimized on test DNAs. The most robust of these, assay P1, amplifies sequences from chr12:7966286–7966484 (P1[A]) and from chr12:8073299–8073582 (P1[B]) (positions according to GRCh37). Figure 3 illustrates how the position of potential single-unit deletion or expansion of this tandem repeat (assuming a simple interunit recombination mechanism of creation) would affect the product ratio P1[B]/P1[A].

In evaluating the P1 assay, 95 Swedish control samples were genotyped in triplicate, and this convincingly revealed five samples for which the P1[B]/P1[A] log_2_ ratio was substantially greater or less than the value of zero (expected for genomes diploid for the “normal” two unit arrangement). Additionally, 12 CEPH DNAs were genotyped in four replicates, and the results were highly similar in each repetition with clear separation of variant and normal samples (Supp. Fig. S2).

### Determining Original Nonallelic Homologous Recombination Recombination Sites

**Figure 4:**
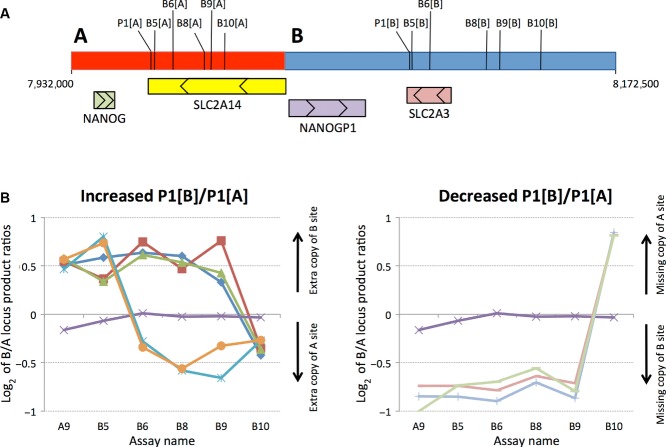
Locating ancestral NAHR events. A: The location of sequences amplified by PRT P1 and pPRTs B5, B6, B8, B9, and B10 are indicated on the tandem repeat (unit A in red, unit B in blue), along with the four genes in the region with arrows showing the direction of transcription. B: The chart-labeled “Increased P1[B]/P1[A]” displays five samples whose previous P1 assay data suggested the presence of an extra copy of the P1[B] sequence on one chromosome, plus a normal sample (purple). Ancestral NAHR events can be taken to have occurred in intervals flanked by assays with markedly different relative abundances of unit B and unit A sequences (see Fig. 3). The blue and orange samples are two Yoruban DNAs, both of which reveal ancestral NAHR events between the sites of assays B5 and B6. The red, dark blue, and green samples are European in origin and reveal NAHR events between markers B9 and B10. The chart labeled “Decreased P1[B]/P1[A]” displays three samples whose previous P1 assay data suggested they were missing the P1[B] site from one chromosome, plus a normal sample (purple). All three non-normal samples are European in origin and reveal additional ancestral NAHR between markers B9 and B10.

**Figure 5:**
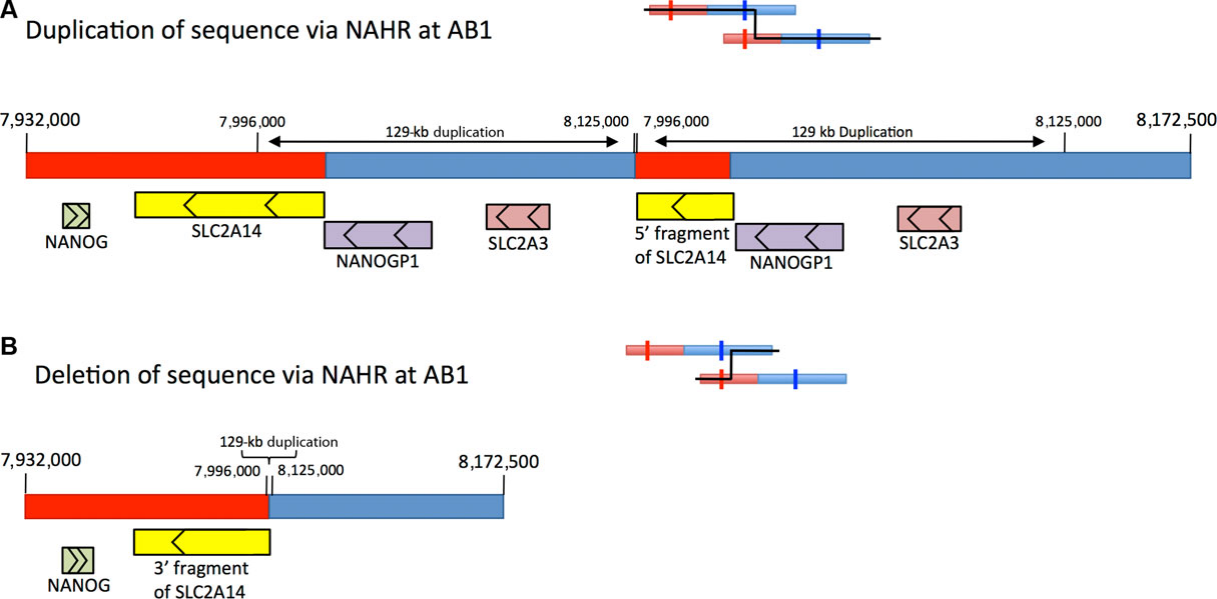
Theoretical products from recombination events at AB1. The two diagrams show the theoretical duplication and deletion products of nonallelic homologous recombination at AB1 (coordinates according to genome reference GRCh37). Red indicates sequence originating from unit A and blue indicates sequence originating from unit B. A: In the chromosomes resulting in duplication, sequence from 7,996,000 to 8,125,000 is duplicated. This causes duplication of *NANOGP1*, *SLC2A3*, and part of *SLC2A14*. B: In chromosomes resulting in deletion, sequence from 7,996,000 to 8,125,000 bp is removed. This causes loss of *NANOGP1*, *SLC2A3*, and part of *SLC2A14*.

Genotyping using P1 across 3,794 UK control samples revealed tight clustering of log_2_ values around those expected for deletions or duplications spanning the P1[B] locus (Supp. Fig. S3). CNVs involving segmental duplications of this size are typically derived by nonallelic homologous recombination (NAHR) [Conrad et al., 2010]. Underlying cross-over sites can be identified by assaying the relative abundances of the tandem duplication sequences in individuals carrying the variant chromosomes. As illustrated in Figure 4, by using a set of “pPRTs” (as explained in *Methods*), specifically designed to quantify abundance ratios for a series of loci across the two tandem repeat elements, we demonstrated that two of two Yoruban HapMap samples with an extra P1[B] copy were generated by NAHR events within an interval of ≈8.8 kb (between B5[A] and B6[A]) in the A unit and ≈6.1 kb in the B unit (between B5[B] and B6[B]). In contrast, three of three European samples with an extra P1[B] copy, and three of three European samples lacking one P1[B] copy, all seem to have been generated by NAHR between pPRTs B9 and B10. Targeted PCR across this latter ancestral breakpoint refined this interval to an 1,100 bp stretch of region, which we termed AB1, shared between the two units (A:7995630–7996700, B:8124315–8125390 [genome build GRCh37])(Supp. Fig. S4). This is by far the main breakpoint in UK samples, in that it was shown to be present in 96.5% of 308 confirmed variant UK DNAs assessed by pPRTs and targeted PCRs. Rearrangements with AB1 breakpoints would directly impact the two glucose transporter genes; *SLC2A3* would be completely deleted or duplicated, and *SLC2A14* would be partially duplicated or deleted (Fig. 5). HGVS nomenclature for the variants is as follows; deletion: chr12.hg19:g.(7995600_7996800)_(8124300_8126400)del, duplication: chr12.hg19:g.(7995600_7996800)_(8124300_8126400)dup).

### CNV Genotyping and Association with RA

To test the 12p13.31 CNV for association with RA, assay P1 was used to examine a Swedish cohort of 2,403 RA cases and 1,269 controls. Genotypes were categorized according to the expected ratios given in Figure 3, with boundaries determined by clustering of P1[B]/P1[A] log_2_ scores. We made the assumption that each individual will have at least one chromosome with the normal allele (likely to be true in almost all subjects, given the low frequency of CNV alleles). As summarized in Table 1, the count of genotypes having a P1[B]/P1[A] log_2_ ratio <−0.75 (i.e., deletion variants that remove the P1[B] locus) is significantly reduced in cases compared with controls (*P* = 0.0012). As expected, the mean log_2_ values for samples with P1[B] deletions were similar between cases (−1.16) and controls (−1.08). For this CNV allele, the odds ratio is 0.442 (95% CI 0.258–0.755), indicating that individuals with a deletion of the region spanning P1[B] are 2–2.5-fold less likely to develop RA. To assess the impact of potential misclassification of the CNV alleles, the boundaries for the P1[B] deletion were varied. The *P* values were seen to remain significant at the 5% level even when extreme thresholds for group classification were applied (Supp. Fig. S5).

**Table 1:** Swedish RA Data

Log_2_ ratio		<0.45 >		
P1[B]/P1[A]	< −0.75	−0.75	>0.45	

	P1(B)		P1(B)	
P1 category	deletion	Normal	duplication	Totals
Case frequency (%)	28 (1.17%)	2,283 (94.95%)	93 (3.88%)	2,403
Control frequency (%)	33 (2.60%)	1,181 (93.06%)	55 (4.34%)	1,269
Odds ratio	0.442^a^ (0.258–0.755)	–	–	–

a χ^2^ *P* value = 0.0012.

### Replication in UK/US Sample Collections

**Figure 6:**
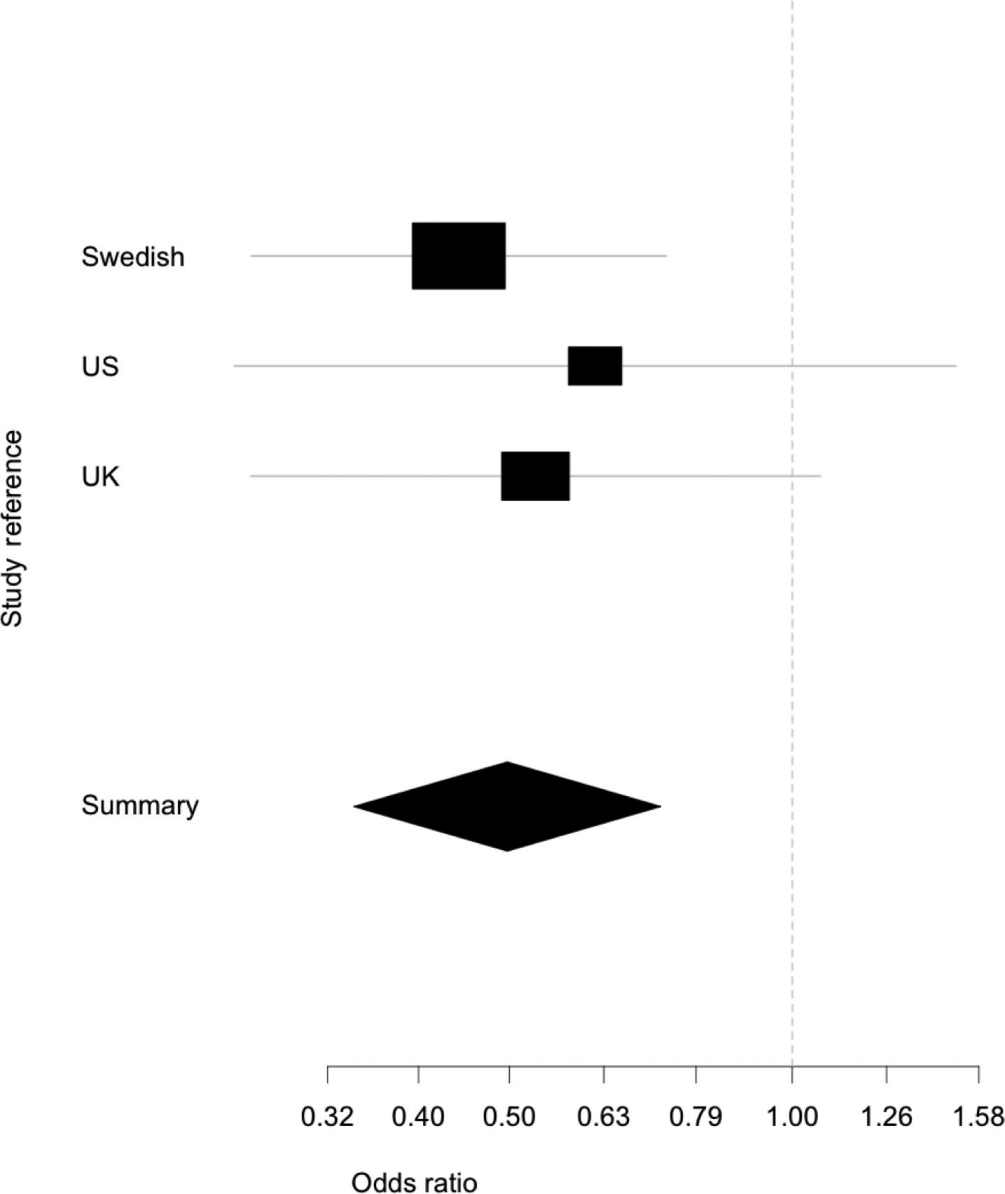
Forest plot of odds ratios for Swedish, US, and UK samples for the deletion variant of the CNV. The diagram, generated by the rmeta package for R 2.16, displays the odds ratios for each population as a box with the 95% confidence intervals indicated by lines. The summary represents the 95% confidence interval of the Mantel–Haenszel combined odds ratio for all three sample collections, the contribution of each population to the combined odds ratio is represented by the size of the odd ratio box for the corresponding population.

A replication study was genotyped for the deletion variant of the CNV using P1 in 9,201 UK samples (7,355 controls and 1,846 cases) and using PennCNV in 2,963 US samples (996 controls and 1,967 cases). Due to power considerations (smaller size of the case or control materials and a lower frequency of deletion variants in UK/US

populations), this disease association analyses considered only the putative etiological deletion variant discovered in the Swedish materials. Association results supported our initial findings completely in terms of direction and effect size of disease risk (Table 2): a decreased frequency of genotypes indicating a deletion was apparent in RA cases compared with controls. This is highly apparent when viewed graphically (Fig. 6). Given the UK and US populations are distinct, both geographically and in method of genotyping we used the Mantel–Haenszel meta-analysis to calculate a pooled odds ratio of 0.559 (95% CI 0.323, 0.966; *P* = 0.036). Importantly, Woolf's test indicated that there is no evidence against homogeneity between the two datasets.

**Table 2:** UK and US RA Data

UK RA data			
Log_2_ ratio P1[B]/P1[A]	<−0.75	>−0.75	
P1 category	P1(B) deletion	Other	Totals
Case	9 (0.49%)	1,837 (99.51%)	1,846
Controls	67 (0.91%)	7,288 (99.09%)	7,355
Odds Ratio	0.533^*^ (0.248–1.107)	**–**	**–**
US RA data			
PennCNV	P1(B) deletion	Other	Totals
Case	11 (0.56%)	1,956 (99.44%)	1,967
Controls	9 (0.90%)	987 (99.10%)	996
Odds Ratio	0.617^a^ (0.237–1.620)	**–**	–

a Combined Mantel–Haenszl odds ratio of 0.559 (*P* = 0.036).

### Genotyping Accuracy Using P1 Assay

The accuracy and robustness of the P1 assay and SNP intensity data are critical to the validity of the disease association we herein report. We applied experimental designs to minimize technical bias, described in *Methods*, and plots of the genotyping measurements (P1[B]/P1[A] ratio, mean LRR, and mean BAF) do not provide any evidence for bias between cases and controls (Supp. Figs. S1 and S6). In addition, we reassessed a subset of 368 samples from the 1,958 UK controls including all 67 samples for which initial genotyping indicated a P1[B] deletion, and a random set of 301 DNAs scored as having neither a deletion nor an insertion. These samples were reexamined by genotyping again using a combination of repeating the P1 assay, using the five independent pPRTs (which had proven ability to detect this CNV in determination of original NAHR events), and direct assessment using the AB1 deletion/duplication-specific assays. Of the 67 deletion samples, 63 were confirmed by AB1 assays, of the remaining four, three were confirmed by 100% replication in the five pPRTs and in two repeats of the P1 assay. Therefore, only one of the 67 deletion samples was found to be a misclassified normal sample—giving a false-positive deletion assignment rate of 1/7,289 (since 7,288 samples were initially scored as nondeletions). Of the 301 normal copy-number samples, none were found to be a misclassified deletion variant sample in either the AB1 assay or in at least two repeats of the P1 assay—giving a false-negative deletion assignment rate of 0/301. These data, representing multiple levels of fully independent assay validation, indicate that the overall misclassification rates are extremely low, and certainly not sufficient to invalidate the discovered disease association.

To provide further quality control for the P1 assay, we compared P1 genotyping results with a set of copy-number assignments generated by a non-PCR-based technology. Specifically, for 1,475 of the Swedish samples genotyped with P1 high-quality Illumina 300k HapMap SNP genotyping data were available, enabling us to call the CNV alleles using the PennCNV algorithm [Wang et al., 2007]. The concordance rate between the P1 assay and PennCNV was 99.7%. The 0.3% discrepancy consists of both the very small error rate described in the UK controls and the error rate of the PennCNV algorithm. Nevertheless, if one were to assume the entire 0.3% originated from the P1 assay, the association in the Swedish study would remain significant.

## Discussion

We have described the discovery of a CNV at 12p13.31 that involves the gain/loss of one net unit (as portions of adjacent units) of a normal two unit tandem repeat, and the association of the deletion allele with RA protection (combined analysis [Sweden, US, and UK] Mantel–Haenszel OR = 0.497 [95%CI 0.341,0.725], *P* = 0.000194). This deletion partially disrupts *SLC2A14* and entirely deletes *SLC2A3* and *NANOGP1*. Since *NANOGP1* is expressed but untranslated and *SLC2A14* is only expressed in the testes, they are not obvious candidates for a direct role in RA. In contrast, the GLUT3 product of *SLC2A3* plays an important role in two key areas relevant to RA: the immune response and chondrocyte function. Related to immune response, activated T-and B-cells, as well as macrophages, are present in RA-affected synovial joints. A 3.5–6-fold increase in the expression of GLUT3 is seen in activated T-and B-cells, and monocyte to macrophage differentiation is associated with an increase in GLUT3 expression. This increased GLUT3 expression in macrophages is maintained after transformation to foam cells and is thought to provide fuel for the immune response, in addition to allowing leukocytes to compete for sugars in low-interstitial glucose concentrations [Fu et al., 2004; Maratou et al., 2007]. Related to chondrocyte function, glucose plays a critical role in chondrocyte metabolism and physiology, and GLUT1, GLUT3, and GLUT9 are all expressed in normal chondrocytes. GLUT3 is essential for facilitated diffusion of glucose into chondrocytes [Mobasheri et al., 2008]. Chondrocytes are involved in RA disease progression through destruction of the extracellular matrix. Evidence for this comes from the exclusive production of the collagen and proteoglycan proteinase MMP-1 by chondrocytes in diseased joints, and from arthritis mouse models where an increased level of cartilage damage was seen when apoptosis of chondrocytes was prevented [Ainola et al., 2005; Barksby et al., 2006; Butler et al., 1997; Otero and Goldring, 2007]. It has also been proposed that chondrocytes themselves may be a source of pro-inflammatory cytokines, which aid joint destruction by increasing the breakdown of tissue and suppressing repair mechanisms. As a result, cartilage is degraded faster than it can be repaired, leading to destruction of the joint [Otero and Goldring, 2007]. With this in mind, we hypothesize that the protective effect of a P1[B] deletion genotype of the 12p13.31 CNV may be due to a decreased capability of individuals with this variant to express *SLC2A3*. This would lead to impairment of the immune response at the synovium, limitation in the ability of chondrocytes to respond to immune signaling and degrade cartilage, or a combination of both mechanisms.

It may be asked why this variant has remained undetected in recent large-scale GWAS involving RA. There are three explanations for this. First, GWAS are designed to test the common variant common disease hypothesis, that is, they rely on LD between common markers and common causal variants (minor allele frequency (MAF) > 5%), and not low-frequency causal alleles. Second, HapMap data comparing SNPs within and neighboring the CNV, and our own data comparing CNV alleles to our previous SNP genotyping, revealed no LD with neighboring SNP alleles with MAF > 5%. This is as expected for multiallelic low-allele frequency CNVs according to published large-scale CNV studies [Conrad et al., 2010]. Third, even if the above two problems did not exist, previous RA GWAS have not employed sufficient samples to have power to detect this locus after correcting for multiple testing.

The effect size of the association we have detected is greater than that for any other RA locus previously described, with the exception of the HLA genes. It is also as large as any previously reported CNV association with any common disease. Additionally, since the mechanism we propose entails a loss-of-function allele that is disease protective, this recommends it as a target for drug development, that is, the inhibition of *SLC2A3* expression (and/or GLUT3 activity) may provide a direct means to protect against RA in the 97%–99% of individuals without the deletion allele. Furthermore, given the tendency for autoimmune disorders to share susceptibility loci, and the role of *SLC2A3* in the immune response, genetic variation in this region could also be important in other immune-related disorders. Finally, we note that GLUT3 has been implicated by altered expression in a number of diseases-including dyslexia, Alzheimer's disease, schizophrenia, and Huntingtons disease, and increased expression of glucose transporters (in particular GLUT1 and GLUT3) is also a characteristic feature of cancer cells [ Macheda et al., 2005; Yamamoto et al., 1990]. We therefore posit that the CNV we have described here may impact the risk of many and various other diseases, and suggest this merits urgent and thorough examination.
