# Supplementary material for: A 129-kb Deletion on Chromosome 12 Confers Substantial Protection Against Rheumatoid Arthritis, Implicating the Gene SLC2A3
Source: Hum Mutat. 2013 Dec 2;35(2):248–56. doi: 10.1002/humu.22471 (PMC3995011; doi:10.1002/humu.22471)
Supplement: Supplementary file 1 [file humu0035-0248-sd1.pdf]

Supp. Table S1. PCR/PRT primers

PCR/PRT	Forward Primer	Reverse Primer A	Reverse Primer B
P1	TATTGCACCTTAACCTCTCCAGC	CCTCACTTCCATACAGCTCTACG	-
B5	AGCAATCCGACTAGGAAGTTGG	CTTGCCATTAGTTGCCTCTGG	GTGAGCTATTGTAGTGCCACTGC
B6	CAGCTAGGGAGTTGCCTCAG	GGAAGAGAGTACTCCTGTGCC	CAGAATCTATGAGGAGCCAAGTG
B8	CTAGGAGTCTGCTCATGCAGG	GAGTGATGTGTCCTCACATGG	CCTCTGCACTCAAGCAATCC
B9	GCTTGCATGAAGTGAAGACAG	GGTAGCCTGGTGATTGTGTCC	GTGGGCATCTGTAATCCAGG
B10	AGGATTCTGCAGAAAGCAGC	AGAGATGGCAGGAGAACAGTC	CTAACACGATGAAGCCTTGTCTC
AF	GCTTGCATGAAGTGAAGACAG	-	-
BF	CAACTCAATGTAAAAGGGAAGGC	-	-
UR	-	TTTGGGAATGGAGGAGCATG	-

A LRR and BAF analysis across Chr12:7884583-8017012 - Deletions

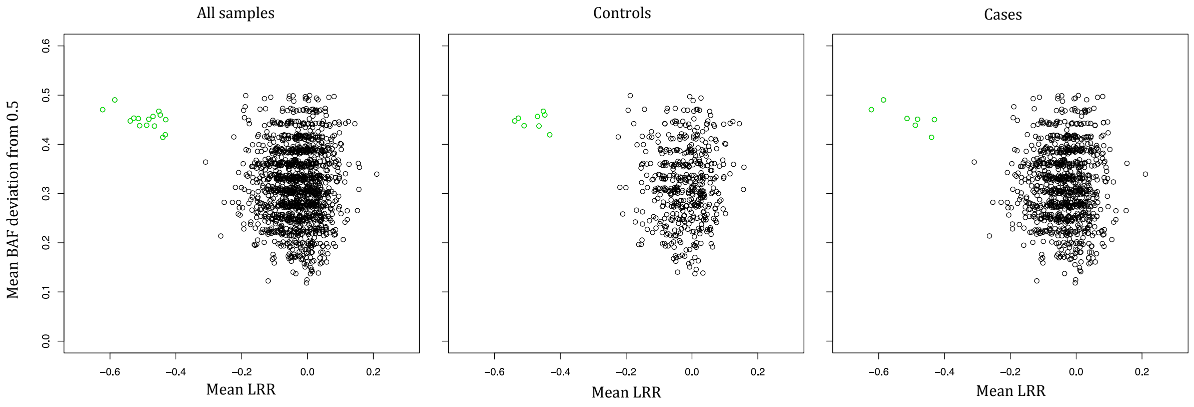

B LRR and BAF analysis across Chr12:7884583-8017012 - Duplications

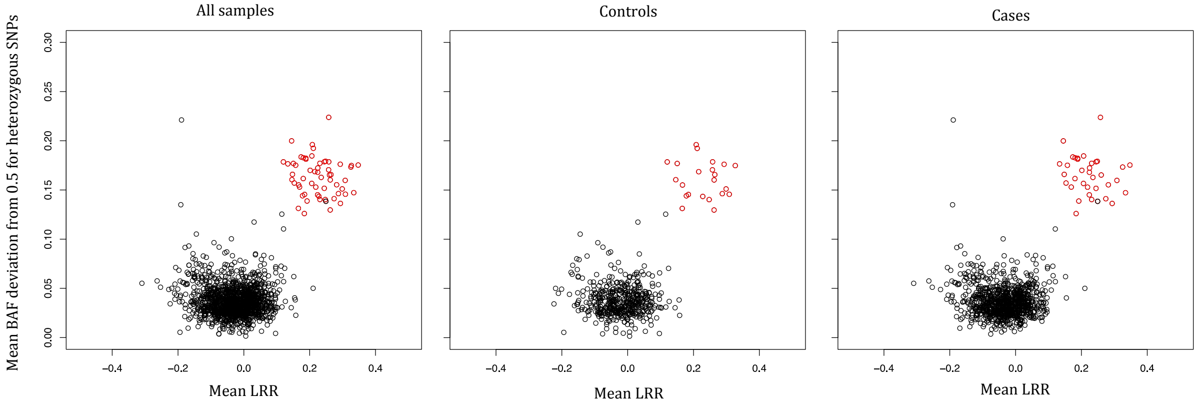

Supp. Figure S1.

A. LRR and BAF analysis across Chr12:7884583-8017012 – Deletions. To visually assess the accuracy of the PennCNV algorithm for calling deletions at chr12:7884583-8017012 we plotted the mean deviation of BAF from 0.5 against mean LRR within the interval (for all samples, and for cases and controls). Samples in green are those determined to have a copy number of 1 by PennCNV. There is a clear distinction between these and the samples with a copy number of 2.

B. LRR and BAF analysis across Chr12:7884583-8017012 – Duplications. For duplications we plotted the mean deviation of BAFs from 0.5 for only heterozygous SNPs against mean LRR within the interval (for all samples, and for cases and controls). Samples in red are those determined to have a copy number of 3 by PennCNV. There is clear clustering between these samples and those with a copy number of 2 with only a minimal overlap.

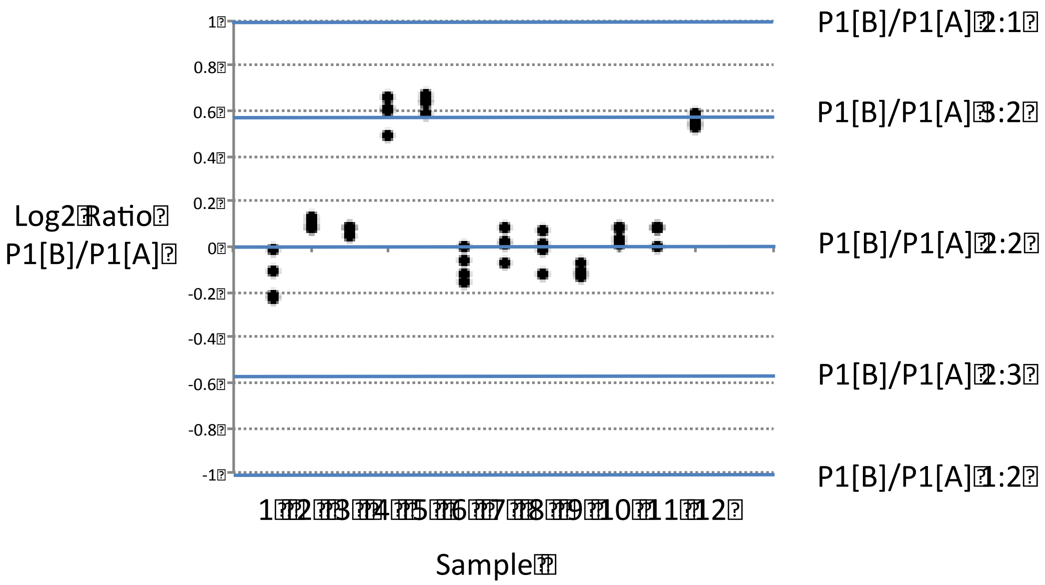

Supp. Figure S2. Reproducibility of the P1 assay. Log_2 ratio plots for 4 replicates of 12 samples used to initially test the P1 assay. Samples 4, 5 and 12 harbour a duplication spanning P1[B] and the remaining samples are normal. Blue lines indicate mid point for predicted variant log_2 ratios.

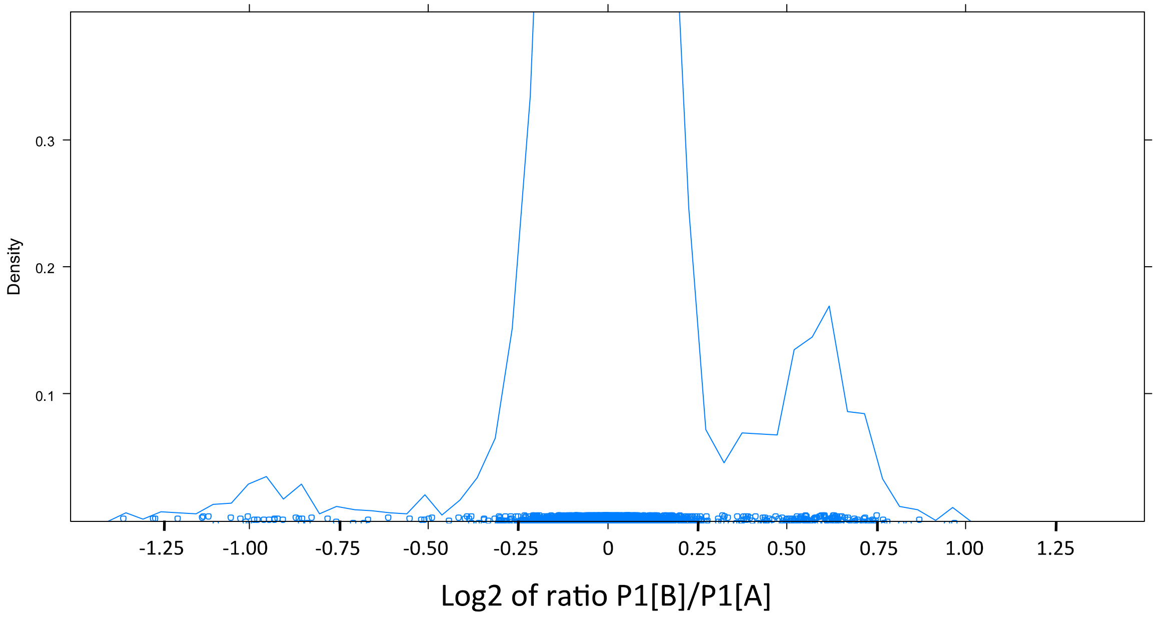

Supp. Figure S3. Density plot (constructed using R 2.7.10) of log2 of P1[B]/P1[A] ratios for 3794 UK samples. The plot has peaks around expected values for duplication of P1[B] (0.58) and deletion of P1[B] (-1).

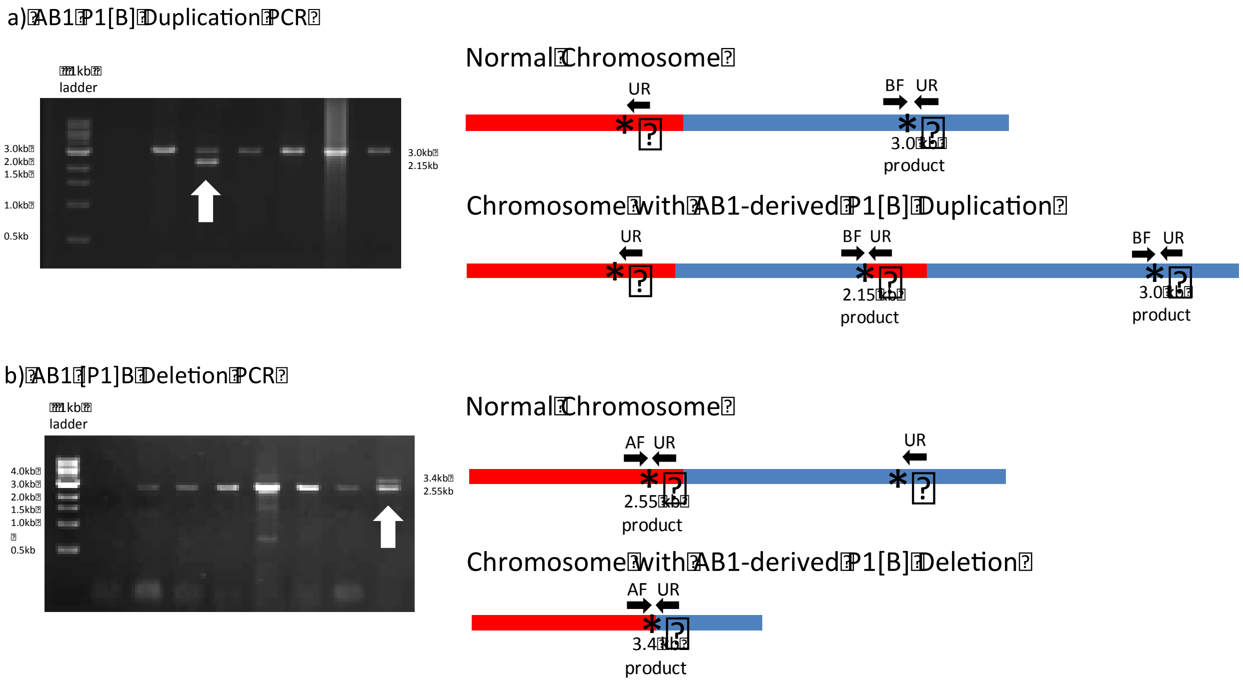

Supp. Figure S4. PCRs to interrogate chromosomes derived from NAHR at the AB1 sequences. The coloured bars represent the units of the tandem repeat. The position of AB1 sequences (asterisk) and primers are indicated. (a) Using primers BF and UR normal chromosomes produce a single band of 3.0 kb. Chromosomes with a P1[B] duplication derived from NAHR at AB1 sequences produce a 3.0 kb from the intact B unit and a 2.15 kb band from the duplicated sequence. (b) Using primers AF and UR normal chromosomes produce a single band of 2.55 kb. Chromosomes with a P1[B] deletion derived from NAHR at AB1 sequences produce a 3.4 kb band.

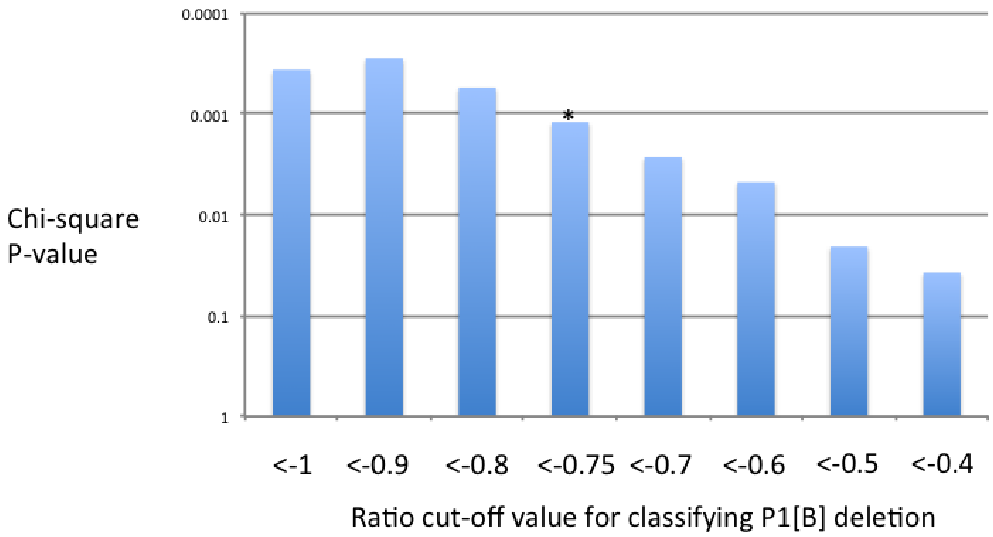

Supp. Figure S5. Log2 ratio values less than -0.75 were classified as P1[B] deletions. For the small possibility that this boundary is inappropriate we also tested different boundaries from -0.4 to -1 and calculated chi-square p-values. In each case the p-value remained significant. The original boundary we selected for association analysis is marked with an asterisk.

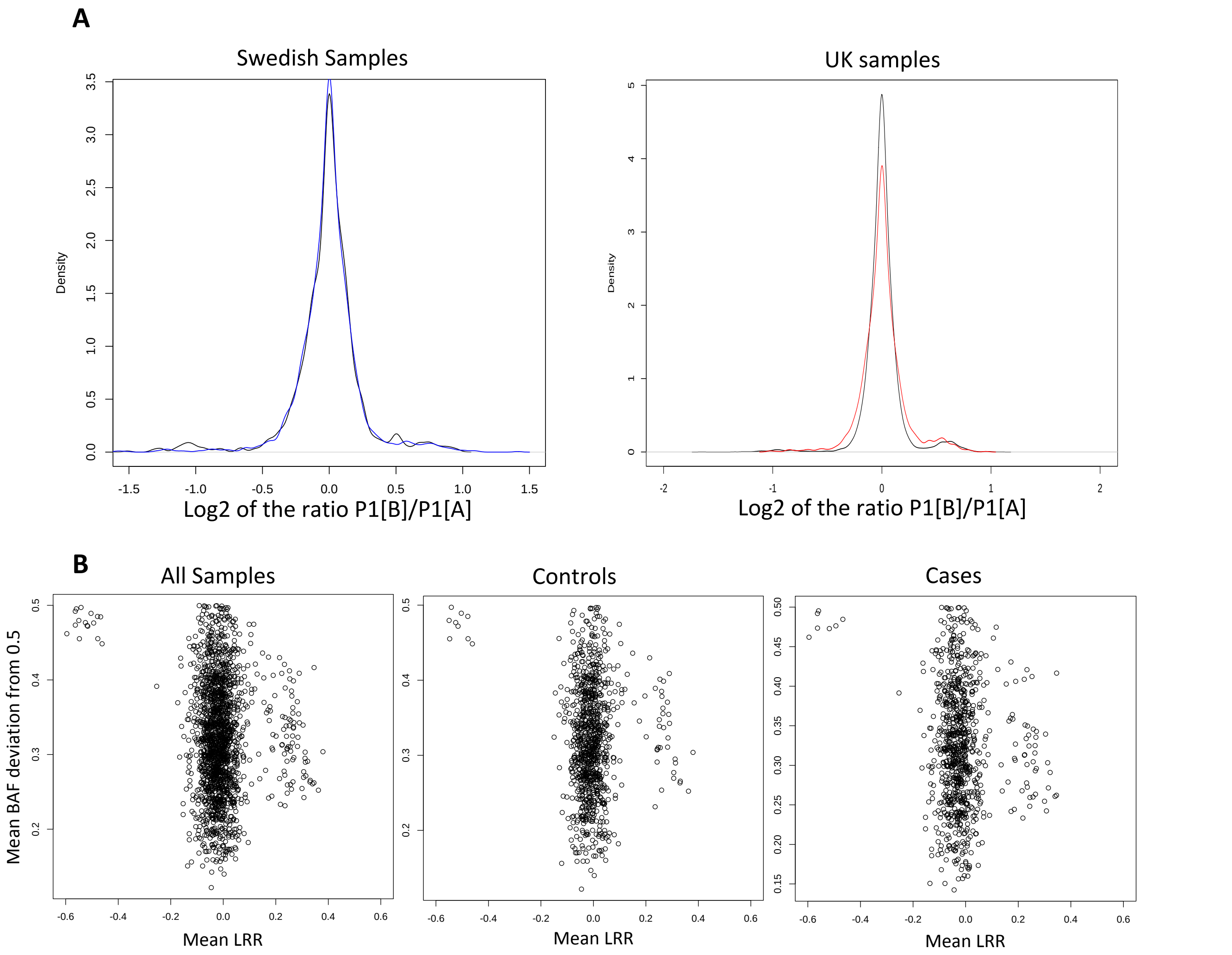

Supp. Figure S6.

A. Density kernel plots showing distribution of P1[B]/P1[A] ratios for the Swedish (controls in black and cases in blue) and UK (controls in black and cases in red) samples. The central portion representing copy number of 2 is the most frequent and the spread is similar, with no skewing, between each group of cases and controls.

B. Scatterplots of mean BAF deviation from 0.5 against mean LRR across the interval chr12:7884583-8017012 for the US samples. The distribution of points is similar between the controls and cases with the deletion group clearly located as a separate cluster in the top left of each plot.
